# Feasibility of a breath robot intervention to reduce sleep problems in posttraumatic stress disorder: protocol for a randomized controlled study

**DOI:** 10.1186/s40814-023-01426-8

**Published:** 2024-02-05

**Authors:** Annett Lotzin, Isabelle Laskowsky

**Affiliations:** 1 Department of Psychology, Institute of Clinical Psychology and Psychotherapy Research, MSH Medical School Hamburg, Hamburg, Germany; 2 Department for Psychiatry and Psychotherapy, University Medical Center Hamburg-Eppendorf, Hamburg, Germany

**Keywords:** Posttraumatic stress disorder, Breath robot, Hyperarousal, Sleep problems, Stress symptoms, Randomized controlled trial, Pilot study

## Abstract

**Background:**

Many patients with posttraumatic stress disorder (PTSD) suffer from sleep problems. Robot-based interventions might be an innovative approach to reduce sleep problems and hyperarousal in PTSD. However, the feasibility and effectiveness of a breath robot in patients with PTSD have never been empirically tested. The aim of this study is to investigate the feasibility of a breath robot to reduce sleep problems and hyperarousal in patients with PTSD.

**Methods:**

This randomized controlled feasibility study will include *N* = 30 adult patients with at least subsyndromal PTSD (PTSD Symptom Scale – Interview-5 (PSSI-5)) according to the Diagnostic and Statistical Manual of Mental Disorders, 5th Edition (DSM-5) and impaired sleep quality (Pittsburgh Sleep Quality Index (PSQI) > 5). Patients with organic sleep disorders or currently in psychotherapeutic treatment are excluded. Study participants are randomized to receive either a 4-week Somnox 2 robot intervention including simulation of human breath or a 4-week Somnox 2 robot intervention without human breath simulation. The primary outcome will be the proportion of randomized participants providing outcome data at post-treatment. We consider a proportion of > 50% to indicate feasibility. Additional feasibility outcomes include eligibility rate, recruitment speed, uptake, retention, treatment adherence, and dropout. Potential outcomes of effectiveness (sleep quality, PSQI; severity of PTSD symptoms, PSSI-5) will be assessed at two time points, before (T0) and after (T1) the intervention. Sleep characteristics (Consensus Sleep Diary (CSD)) are measured daily.

**Discussion:**

This study is the first to investigate the feasibility of a novel breath robot intervention for reducing sleep problems and hyperarousal in PTSD patients, with effectiveness considered as a secondary outcome. If feasible and effective, the use of a breath robot could be a nonintrusive and flexible intervention to supplement psychotherapy or to bridge treatment gaps.

**Trial registration:**

DRKS, DRKS00031063. Registered on 10/012023.

## Introduction

One-third of the general population experiences a potentially traumatic event such as a car accident, rape, or physical abuse at least once in their life [23]. Approximately 12% of those affected develop posttraumatic stress disorder (PTSD), a severe mental disorder associated with psychological, social, and functional impairments [23]. Patients with PTSD show constantly increased stress levels and hyperarousal [6], both of which are associated with sleep disruptions [2]. Eight out of ten patients have difficulty falling or staying sleeping [6]. PTSD is therefore linked to greater sleep latency, sleep fragmentation, and night-to-night sleep variability, as well as to reduced sleep efficiency (for an overview see [14]). Such sleep problems associated with PTSD impair mental health [13] and quality of life [8, 26].

Slowing the breath can reduce stress in PTSD patients by affecting the autonomic nervous system [24]. Breathing techniques are regularly applied in the psychological treatment of PTSD to promote relaxation and reduce hyperarousal. While evidence-based psychotherapies exist for PTSD-related sleep problems [30, 31], their availability is limited [18]. In Germany, approximately 40% of the patients in need of psychotherapeutic treatment wait longer than 6 months before they start treatment [11]. The application of robot-assisted interventions may help to reduce these treatment gaps. Compared to psychotherapeutic treatment, robotic interventions require less time, space, and staff. Social robots that interact with humans could replace or enhance psychotherapeutic interventions [7, 9]. Robotic interventions could be used as an adjunct to psychotherapy or to bridge waiting times. However, robots have not yet been used in PTSD treatment.

First, research is needed to examine the feasibility and effectiveness of robotic breath interventions on sleep problems in PTSD patients. Therefore, the primary aim of this study is to examine the feasibility of using a breath robot (Somnox 2) in patients with PTSD. The secondary aim is to examine the potential effectiveness and the users’ experiences with the breath robot intervention to reduce sleep problems and hyperarousal.

## Methods

### Trial design

The study is a randomized controlled, investigator-blinded feasibility study with two groups. After study inclusion, participants are randomly assigned to a 4-week breath robot intervention group (active treatment: daily use of a robot stimulating human breathing) or a control group (sham treatment: daily use of a robot without breathing stimulation).

### Study setting

The study is conducted at the Institute of Clinical Psychology and Psychotherapy at the Department of Psychology at MSH Medical School Hamburg. Participants will be recruited from psychotherapeutic outpatient units and via announcements on widely accessed websites (e.g., eBay Classified Ads).

### Study procedure

**Fig. 1:**
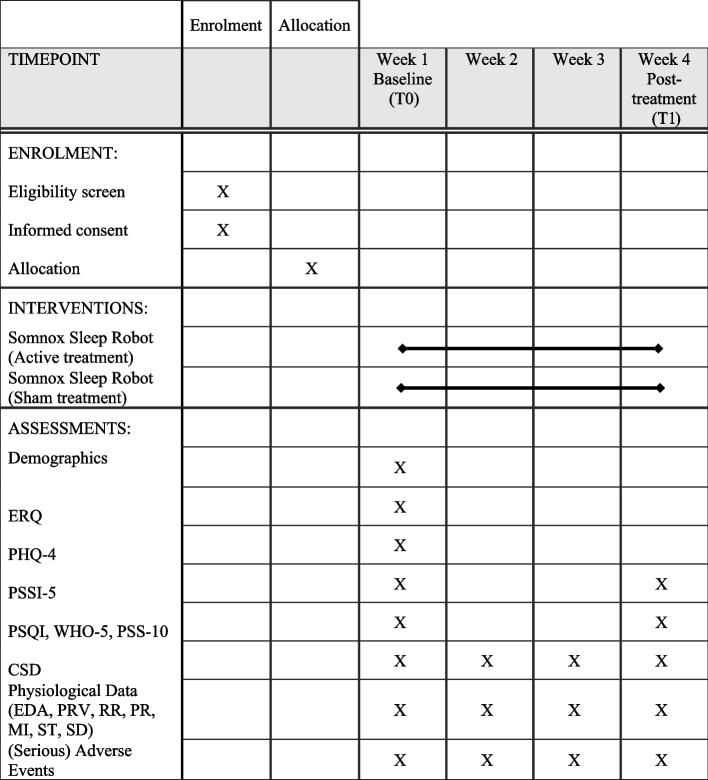
Schedule of enrollment, intervention, and assessment. Notes : ERQ, Emotion Regulation Questionnaire; PHQ-4, Patient Health Questionnaire-4; PSSI-5, PTSD Symptom Scale – Interview for DSM-5; PSQI, Pittsburgh Sleep Quality Index; WHO-5, World Health Organization Well-Being Index; PSS-10, Perceived Stress Scale-10; CSD, Consensus Sleep Diary; EDA, electrodermal activity; PRV, pulse rate variability; RR, respiratory rate; PR, pulse rate; MI, movement intensity; ST, skin temperature; SD, sleep detection

Potential participants are invited by the research personnel to attend an eligibility assessment at one of two study locations of MSH Medical School Hamburg. The study personnel inform interested participants about the study and the procedures and provide the opportunity to ask questions. Potential participants receive a study sheet that provides information about the study objectives, study procedures (treatment and assessment), eligibility criteria, random allocation process, type of treatment (including benefits and risks), compensation for study participation, right of study withdrawal, data flow and security, and contact information. Subsequently, potential participants participate in a 2-h baseline assessment (T0), during which the study staff review the eligibility criteria and assess the baseline data. Eligible participants who agreed to participate sign the informed consent for the study. The study staff instruct participants on how to use the sleep robot, the technical equipment to measure digital biomarkers, and how to complete the daily sleep assessments. During the 4-week study period, participants apply the robot for at least 30 min daily before bedtime and, if possible, throughout the day. Participants will be instructed to complete the sleep diary (CSD) daily on a smartphone no later than 1 h after waking. To collect digital biomarkers, participants are instructed to wear the (EmbracePlus; [10]) day and night, except when the device needs charging. A research assistant calls the participants once a week to provide support in using the breath robot and wearable device and to clarify questions, if needed. Post-treatment (T1), the participants attend a 2-h assessment with the study staff to reassess the variables measured at T0 (Fig. 1).

### Participants and eligibility criteria

Participants are included according to the following criteria: (1) 18 years of age or older; (2) diagnosis of at least subsyndromal PTSD (i.e., trauma exposure as defined in criterion A of the DSM-5 PTSD diagnosis, probable PTSD (PSSI-5 cutoff ≥ 23)), at least one intrusion symptom and one arousal / reactivity symptom (PSSI-5); and (3) impaired sleep quality (PSQI > 5). Participants are excluded according to the following criteria: (1) organic sleep disorder (e.g., sleep apnea, narcolepsy, restless legs syndrome) and (2) participation in psychotherapeutic treatment during the study period, except for the initial psychological consultations or probationary sessions to plan psychotherapeutic treatment.

### Intervention

**Fig. 2:**
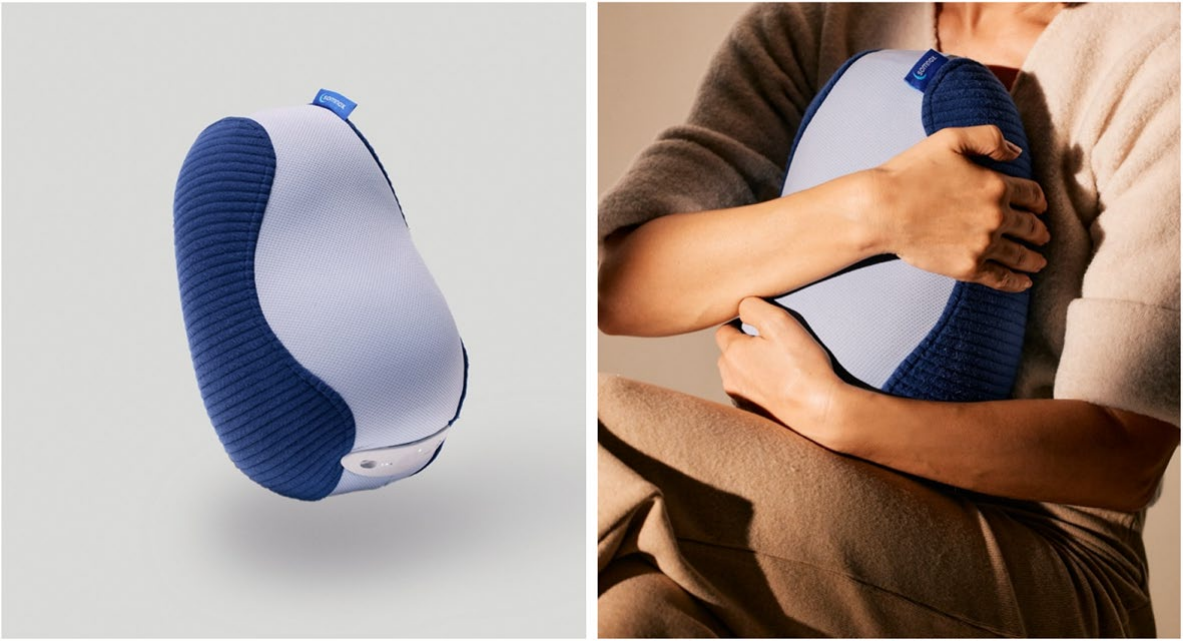
The Somnox 2 sleep robot. Note : Copyright 2023 by Somnox. Used with permission

The Somnox robot [33] was developed at the Robotics Institute of the Delft University of Technology in the Netherlands to improve sleep. The current version, Somnox 2, has the form of a bean-shaped cushion to hug and simulates human breathing (Fig. 2).

Somnox 2 is designed to reduce the user’s breath rate. When hugging, the robot detects the breath rate of the user and simulates a human breath with a slightly lower rate compared to the user. It is assumed that the users synchronize their breath with the breath of the robot. The aim of the robot breath intervention is to slow down breathing to promote relaxation and thus good sleep.

The participants of the intervention group are instructed to hold the sleep robot in their arms (i.e., to create physical contact) so that the breathing of the robot can be sensed (Fig. 2). They receive written and verbal instructions on how to use the robot and its functions. The robot has a soft surface that is pleasant to touch. The participants have the choice to adjust the breathing settings (e.g., breath rate) manually via the Somnox mobile app while using the Somnox 2. They have the option to play soothing music or nature sounds via the app. Over the 4-week period, participants are asked to hug the robot daily for at least 30 min in bed until they fall asleep and to resume this after any unwanted awakenings before sleeping. They are also instructed to use the robot whenever they like throughout the day.

### Control group

The participants in the control group are instructed to use the robot daily for at least 30 min in bed until they fell asleep and to resume this after any unwanted awakenings before sleeping. They are also instructed to use the robot whenever they like throughout the day. In the control condition, the breathing and relaxation music functions of the Somnox 2 are deactivated.

### Outcomes

#### Primary outcome

We evaluate the feasibility of the outcome data collection, focusing on randomized participants providing data at post-treatment (T1) after a 4-week Somnox 2 robot intervention with human breath simulation, or after a 4-week Somnox 2 robot intervention without breath simulation. Feasibility, set at > 50%, applies equally to both groups. This criterion considers potentially high dropouts in individuals with mental health conditions and the technical complexity of the study (e.g., using a wearable for 4 weeks).

#### Secondary outcomes

##### Feasibility

Eligibility rate (proportion of included participants out of all screened individuals), recruitment speed (number of participants recruited in 6 months), uptake (proportion of participants who started the robot intervention out of those randomized in the intervention group), retention (proportion of participants in the intervention arm that received at least 20 robot sessions), treatment adherence (proportion of days the robot was used, average daily robot use in minutes), and dropout rate (rate of participants who were randomized but terminated study before completion of T1).

##### Effectiveness

Differences in sleep quality (Pittsburgh Sleep Quality Index, PSQI; [4]), severity of PTSD symptoms (PTSD Symptom Scale – Interview for DSM-5, PSSI-5; [12]), perceived distress (Perceived Stress Scale-10, PSS-10; [25]), and well-being (World Health Organization Well-Being Index, WHO-5; [32]). Changes in sleep characteristics over the 4-week study period (e.g., time of falling asleep; Consensus Sleep Diagry, CSD; [5]), are measured daily. Changes in digital biomarkers (electrodermal activity, pulse rate, respiratory rate, peripheral skin temperature, movement intensity, and sleep) are continuously assessed during the study period using a wearable (EmbracePlus; [10]).

##### Participants’ subjective experience

Patients’ views on the feasibility and effectiveness of the intervention and mechanisms of action for positive and/or negative effects are captured by a self-constructed qualitative interview at T1.

##### (Serious) adverse events

The study staff inquire about possible adverse events in the weekly support calls.

### Measures

#### Baseline assessment (T0)

Sociodemographic and health-related data (e.g., medication, psychotherapeutic treatment) are assessed at T0 using self-constructed items. Depression and anxiety symptoms are measured at T0 using the Patient Health Questionnaire-4 (PHQ-4; [21]). The PHQ-4 consists of four items: two items capture depressive symptoms, and two items capture anxiety symptoms. Participants are asked to rate how often they have been bothered by each symptom over the past 2 weeks using a Likert scale ranging from 0 (*not at all*) to 3 (*nearly every day*). The PHQ-4 has demonstrated strong reliability and validity as a brief screening tool for assessing symptoms of depression and anxiety (e.g., [21]).

Differences in emotion regulation are measured at T0 with the Emotion Regulation Questionnaire (ERQ; [15]). The ERQ consists of ten items, with six items assessing cognitive reappraisal and four items assessing expressive suppression. Participants rate their agreement with each item on a scale ranging from 1 (*strongly disagree*) to 7 (*strongly agree*). The ERQ has demonstrated consistent reliability and validity in clinical contexts (e.g., [15]).

#### Assessment at baseline (T0) and post-treatment (T1)

Sleep quality and disturbances in the past 2 weeks are measured with the Pittsburgh Sleep Quality Index (PSQI; [4]). The PSQI consists of 19 items grouped into seven component scores, each reflecting a different aspect of sleep quality: subjective sleep quality, sleep latency, sleep duration, sleep efficiency, sleep disturbances, use of sleep medication, and daytime dysfunction. The directionality and interpretation of scores vary across items. A total score ranging from 0 to 21 can be calculated. Higher scores indicate poorer sleep quality. The PSQI has shown good reliability and validity across several studies (e.g., [1, 4]).

PTSD symptoms are measured using the 24-item semistructured PTSD Symptom Scale – Interview for DSM-5 (PSSI-5; [12]). The PSSI-5 assesses PTSD symptoms according to DSM-5. The interview is divided into four sections: re-experiencing, avoidance, changes in cognition and mood, increased arousal and reactivity, and distress and interference. Item scores range from 0 (*not at all*) to 4 (*6 or more times a week/severe*), the ratings refer to the past month. The interviews were conducted by two research assistants which underwent comprehensive training in the interview procedures. The PSSI-5 demonstrated good validity and reliability in a sample of adults who had experienced DSM-5 Criterion A traumatic events [12].

Stress symptoms are assessed using the German adaptation of the Perceived Stress Scale-10 (PSS-10; [25]). PSS-10 items range from 1 (*never*) to 5 (*very often*), with higher scores indicating higher perceived stress. The PSS-10 has shown good reliability and validity in populations of university students and adults [20].

Well-being is measured by the World Health Organization Well-Being Index (WHO-5; [32]). The WHO-5 includes five items measuring positive psychological well-being. Each item is rated from 0 (*not present*) to 5 (*constantly present*) on a 6-point Likert scale. The WHO-5 has been shown to be a reliable and valid measurement tool in a variety of settings (e.g., [16, 27, 32]).

##### Daily assessments

Sleep characteristics are measured daily in the morning after waking up using the Consensus Sleep Diary (CSD; [5] over 4 weeks). Nine items assess the time of bedtime, time of sleep attempt, time needed to fall asleep, number of awakenings during sleep, total duration of waking up, time of final waking up, wake up time, and sleep quality. The CSD has shown good psychometric properties regarding utility and validity in sleep disorders [22].

##### Continuous assessments

Digital biomarker data are recorded continuously over 4 weeks by using the Empatica EmbracePlus wearable. The medical device was developed for research purposes and collects physiological biomarkers such as electrodermal activity, pulse rate, respiratory rate, peripheral skin temperature, movement intensity, and sleep detection.

### Sample size

We will include *N* = 30 participants. Given that the primary study aim is to assess feasibility, no formal sample size calculation was conducted, aligning with the recommendation that feasibility studies do not require sample size estimates [19]. The number of participants was limited to *N* = 30 since this is the minimum sample size needed to estimate a parameter for testing group differences [3]. This sample size meets the recommendation from Whitehead et al. [34] of a minimum of 15 participants per group when estimating the variance for a sample size calculation for a future trial designed with 90% power, two-sided 5% significance, and a medium (0.5) effect size. Interim analyses are not planned.

### Randomization

The allocation schedule for the random assignment of the individuals to two groups was generated by the randomization software DatInf RandList version 1.2 by a researcher not involved in other parts of the study. The generation of the randomization sequence took place prior to the recruitment of participants. *N* = 30 participants are randomly assigned to one of the two groups (i.e., robot intervention with breath simulation versus robot intervention without breath simulation) in a ratio of 1:1 using permuted blocks of random sizes between 2 and 6. Each random number is separately stored in a sequentially numbered, opaque and sealed envelope. Only the unblinded research assistant responsible for the randomization has access to the randomization envelopes. After the eligibility of a participant for the study has been ensured, the participant is included in the study by a research assistant, and the next available randomization number is assigned to the participant in an ascending order.

### Blinding

The study personnel assessing the study outcomes and conducting the data analysis are blinded to the treatment allocation. Participants could not be blinded due to the nature of the study.

### Statistical methods

Due to the pilot character of the study, the main analysis will be descriptive. Descriptive statistics will be computed for all outcomes assessed at T0 and T1 or daily, separately for the two groups. The means and standard deviations will be calculated for the continuous variables, and frequencies and percentages will be calculated for categorical variables. Cohen’s *d* effect sizes will be calculated for the outcomes to provide an indication of the magnitude of observed differences. We will also examine the confidence intervals around the point estimates for relevant outcomes, offering a more nuanced interpretation of the results.

## Discussion

This pilot study will be, to our knowledge, the first randomized controlled study on the feasibility and effectiveness of a breath robot (Somnox 2) intervention to reduce sleep problems in patients with PTSD. The findings may be of particular importance as sleep problems are common in PTSD.

Few studies have examined the improvement of sleep through robotic interventions in patients with dementia. Recently, a systematic review and meta-analysis on the effects of robotic interventions to improve sleep identified four controlled studies among older adults, most of whom were diagnosed with dementia [28, 29]. The interventions had no significant effects on sleep duration compared to the control conditions. However, most of the trials were of low study quality, and all examined robot interventions not designed to improve sleep. Two additional studies not considered in the review examined the effects of a breath robot designed to improve sleep. One study examined a robotic breath intervention with *N* = 36 premature infants [17]. Infants used a “breathing” teddy bear that was adapted to the breathing pattern of the infants. The use of the breath robot for 2 weeks resulted in improved sleep quality post-intervention. One pilot RCT examined the effects of a 3-week breath robot intervention (Somnox robot) in *N* = 44 adults with insomnia [29]. No diferences in insomnia symptoms were found between the intervention and wait-list control groups. However, the study was small, and not all patients fully adhered to the intervention. The planned pilot study outlined in this protocol will add first evidence on the feasibility and effectiveness of a breath robot intervention among patients with PTSD.

A methodological strength of this study is that we follow good practice recommendations for pilot and feasibility studies [19]. We also consider the Standard Protocol Items: Recommendations for Interventional Trials (SPIRIT) checklist as a guide for reporting this study protocol. Another strength is that we diagnose PTSD using a structured clinical interview. For clinical outcomes, we use validated self-report measures that we combine with objective biomarkers assessed continuously over 4 weeks in the home environment of the patients. However, most data collected include self-reported data, which can introduce measurement error.

Another strength of this study is the inclusion of a control group. We investigate the effectiveness of the daily use of a breath robot with the daily use of the same robot without breath simulation. Such a study design allows us to investigate whether the breathing of the robot  – which is assumed to be the main mechanism of action  – is crucial for the effect of the robot intervention, if present. However, this study design cannot provide information about whether the overall robot intervention, including daily hugging of the robot with a nice texture, perception of the robot’s breathing, and listening to relaxing music, will more greatly reduce sleep problems in PTSD patients compared to no intervention.

Although both study groups receive a similar intervention and instruction, participants cannot be blinded to whether the robot is breathing. The study is therefore susceptible to participant effects. However, the study personnel assessing the results are blinded, as is the person performing the statistical analysis. Another shortcoming of this study could be seen in the fact that 30 participants are too few to detect small effects. However, this study is primarily designed to assess feasibility and not to detect intervention effects. We also followed recommendations for determining sample size in pilot trials [3, 34]. The study is further limited to examining the feasibility and effectiveness over a 4-week period, as we do not conduct follow-up measurements.

Despite these limitations, we assume that this feasibility study will gain novel insights into the application of a breath robot in PTSD patients with sleeping problems. As no previous study has been conducted on this research question, it is unclear whether patients with PTSD will accept the intervention and whether the intervention will have any positive or negative effect on sleeping problems or arousal. However, if the breath robot will be feasible and effective in this patient group, the intervention has the potential to significantly improve the treatment of PTSD. Given the shortage of psychotherapy and the often long wait times for psychotherapy, it is critical to develop alternative treatment options. Breath robots such as the Somnox 2 may be more readily available, cost less, and can be used more flexibly at home compared to psychotherapy. However, based on current empirical knowledge, there is no evidence that breath robots are effective in reducing sleep problems in patients with PTSD.

## Data Availability

The data sets generated or analyzed during the current study are not publicly available because the trial is ongoing. After the publication of the results of this study, the respective anonymized data will be made available by the corresponding author upon reasonable request.

## Abbreviations

DSM-5: Diagnostic and statistical manual of mental disorders
PTSD: Posttraumatic stress disorder
EDA: Electrodermal activity
PRV: Pulse rate variability
RR: Respiratory rate
PR: Pulse rate
MI: Movement intensity
ST: Skin temperature
SD: Sleep detection
RCT: Randomized controlled trial
ERQ: Emotion Regulation Questionnaire
PHQ-4: Patient Health Questionnaire-4
PSSI-5: PTSD Symptom Scale – Interview for DSM-5
PSS-10: Perceived Stress Scale-10
WHO-5: World Health Organization Well-Being Index
CSD: Consensus Sleep Diary
PSQI: Pittsburgh Sleep Quality Index
SPIRIT: Standard protocol items: recommendations for interventional trials
